# Temporal proximity to the elicitation of curiosity is key for enhancing memory for incidental information

**DOI:** 10.1101/lm.052241.120

**Published:** 2021-02

**Authors:** Charlotte Murphy, Vera Dehmelt, Andrew P. Yonelinas, Charan Ranganath, Matthias J. Gruber

**Affiliations:** 1 Cardiff University Brain Research Imaging Centre (CUBRIC), School of Psychology, Cardiff University, Wales CF24 4HQ, United Kingdom; 2 Department of Psychology, University of California at Davis, Davis, California 95616, USA; 3 Center for Neuroscience, University of California at Davis, Davis, California 95616, USA

## Abstract

Curiosity states benefit memory for target information, but also incidental information presented during curiosity states. However, it is not known whether incidental curiosity-enhanced memory depends on when incidental information during curiosity states is encountered. Here, participants incidentally encoded unrelated face images at different time points while they anticipated answers to trivia questions. Across two experiments, we found memory enhancements for unrelated faces presented during high-curiosity compared with low-curiosity states, but only when presented shortly after a trivia question. This suggests processes associated with the elicitation of curiosity—but not sustained anticipation or the satisfaction of curiosity—enhance memory for incidental information.

Curiosity has widely been assumed to benefit memory, with theories postulating that curiosity is a motivational state that stimulates information seeking to reduce uncertainty (Berlyne 1966; Loewenstein 1994; Litman et al. 2005; Kidd and Hayden 2015; Gottlieb and Oudeyer 2018; Gruber and Ranganath 2019). Research that addresses the relationship between curiosity and learning has typically used a trivia paradigm in which participants are tested on memory for answers to trivia questions that elicit different levels of curiosity (e.g., Kang et al. 2009). These studies demonstrate that memory of trivia answers is higher for questions that elicited high levels of curiosity (referred to here as curiosity-enhanced memory) (e.g., Kang et al. 2009; Gruber et al. 2014; McGillivray et al. 2015; Marvin and Shohamy 2016; Fastrich et al. 2018; Wade and Kidd 2019).

Evidence also demonstrates that curiosity significantly enhances memory for incidental information. For example, Gruber et al. (2014) presented an incidental face image in the middle of the anticipation period between eliciting curiosity (via the presentation of a trivia question) and satisfying curiosity (via the presentation of an answer to a trivia question). Memory for the incidental face image was higher when participants anticipated answers with high compared with low curiosity. Therefore, states of high curiosity not only improve learning for topics that pique an individual's curiosity, but a high-curiosity state can also improve memory of information beyond the target of a person's curiosity (for further replications, see Gruber et al. 2014; Galli et al. 2018; Stare et al. 2018; Fandakova and Gruber 2020). However, it is not clear how memories are enhanced for incidental information during curiosity states.

Neuroimaging research has shown that curiosity states increase activity within the dopaminergic circuit (Kang et al. 2009; Gruber et al. 2014; Duan et al. 2020) and thereby benefit hippocampus-dependent memories for curiosity target and incidental information (see the Prediction, Appraisal, Curiosity, and Exploration [PACE] framework for a theoretical framework) (Gruber and Ranganath 2019). Importantly, in one fMRI study (Gruber et al. 2014), we showed that the neural dynamics predicting curiosity-related memory enhancements for incidental images were evident when curiosity was elicited (i.e., during the presentation of a trivia question associated with high curiosity). As activation in the dopaminergic circuit increases phasic dopamine release in the hippocampal memory system (Lisman and Grace 2005; Kang et al. 2009; Rossato et al. 2009; Düzel et al. 2010; Shohamy and Adcock 2010), we theorize that curiosity-enhanced memory for incidental information will be higher when the incidental information is presented in close proximity to when curiosity is elicited. Alternatively, arousal-biased competition theories stipulate that arousal, potentially elicited via trivia stimuli, suppresses competing nontarget mental representations (e.g., such as incidental faces) in favor of goal-relevant stimuli (Mather and Schoeke 2011; Mather et al. 2016). Arousal-based theories would therefore suggest there would be a decrease in memory for incidental items in close proximity to the elicitation of curiosity where biasing of target information would be greater.

In order to further disentangle whether early rather than late processes during curiosity states affect memory for incidental information, we performed two behavioral experiments building on previous work using the trivia paradigm. In experiment 1, we used a between-subjects design in which the incidental face image was shown either early or late during the anticipation period (i.e., either subsequently after the presentation of the trivia question or immediately preceding the trivia answer). In experiment 2, we used a within-subjects design and further interrogated the findings of experiment 1 by spanning the presentation of unrelated face images across the whole anticipation period (i.e., at one of four possible time points). This allowed us to investigate whether a linear relationship existed between the magnitude of curiosity-enhanced memory of incidental information and the time point at which it was presented.

Across two experiments, participants underwent a three-stage paradigm with (1) a screening phase, (2) a study phase, and (3) a surprise recognition test phase for incidental face images. During the screening phase, we obtained an equal number of low- and high-curiosity questions for which participants did not know the answers (for details, see Fig. 1A; Supplemental Material, section 1.2). In the subsequent study phase (Fig. 1B–D), the selected trivia questions were randomly presented followed by an anticipation period that preceded the presentation of the associated trivia answer. During the anticipation phase, a crosshair was presented that was replaced by an image of an emotionally neutral unrelated face.

**Figure 1.**
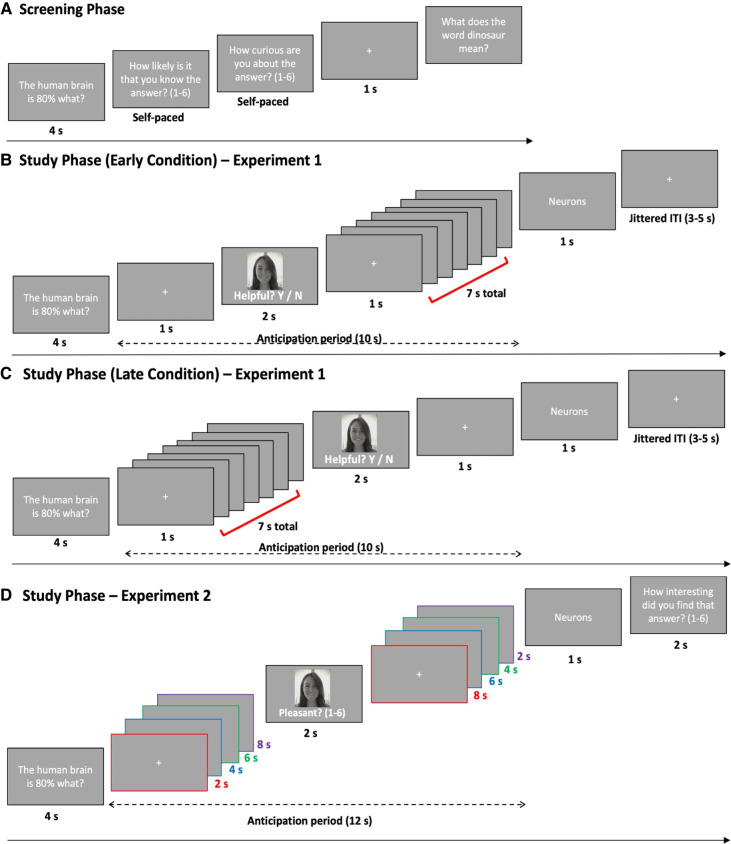
Experimental design. (*A*) During the Screening phase, trivia questions were randomly selected from a pool of trivia questions (the trivia stimuli are available online at OSF https://osf.io/he6t9). Participants rated their prior knowledge and curiosity for trivia questions on a six-point scale. In experiment 1, the screening phase lasted until the participant selected 56 low-curiosity trials (pressed 1, 2, and 3) and 56 high-curiosity trials (pressed 4, 5, and 6) of which they do not have prior knowledge (112 trials in totals). In experiment 2, this lasted until 64 low-curiosity trials and 64 high-curiosity trials were selected (128 trials in total). (*B*,*C*) Study phase experiment 1: Participants encoded trivia questions (4 sec), followed by a 10-sec anticipation period (depicted by the dashed line). Using a between-subjects design, participants were preallocated into early or late conditions. For the early condition an emotionally neutral face (incidental item) is presented after 1 sec (*B*) and for the late condition an emotionally neutral face is presented after 7 sec (*C*). Participants rated (yes/no) whether this particular person would be knowledgeable about the trivia topic and could help them figure out the answer. After the anticipation period, the trivia answer was presented (1 sec). The end of a trial is denoted by a white fixation on a gray background (3–5 sec jittered intertrial interval [ITI]). (*D*) Study phase experiment 2: Participants were presented with trivia questions (4 sec), followed by a 12-sec anticipation period (depicted by the dashed line). After a pseudorandom period of time (2, 4, 6, or 8 sec) an emotionally neutral face (incidental item) was presented in the anticipation period (2 sec), and participants were to rate how pleasant they find the image on a scale from 1 (“not at all pleasant”) to 6 (“extremely pleasant”). The anticipation period then continued until a total of 12 sec had passed since the presentation of the trivia question. The color boxes are for explanatory purposes only and denote the four timing combinations of when the incidental face could be presented. For example, if a fixation period lasted 4 sec before the incidental face image was presented (2 sec) the remaining anticipation period lasted 6 sec (12 sec in total)—highlighted by the blue boxes. After the anticipation period, the trivia answer was presented (2 sec). The end of a trial is denoted by a white fixation on a gray background (2‐‐ to 4-sec ITI). For both experiments, the study phase was divided into four blocks.

In experiment 1 (N = 61) (see Supplemental Material, section 1.1), the face image was shown either 1 sec after question offset (early condition) (Fig. 1B) or 7 sec after question offset (late condition) (Fig. 1C). During the presentation of the face, participants had to give a yes/ no response as to whether this particular person would be knowledgeable about the trivia topic and could help them figure out the answer (cf., Gruber et al. 2014). This encoding judgment was used to ensure that faces were likely to be encoded with a similar level of attention across both curiosity conditions. Following the encoding task, a surprise recognition memory test for the faces was administered. The recognition test occurred ∼10 min after the end of the study phase. Participants were tested with a six-way recognition judgment to dissociate between recollection- and familiarity-based recognition of incidental face images (see Supplemental Material, section 1.3).

To investigate curiosity-enhanced memory for incidental face images in experiment 1, we used a two-way mixed-effects ANOVA to test if curiosity (two levels: high vs. low) was positively associated with better memory performance and whether the time point of face presentation (two levels between-subjects: early vs. late) interacted with the potential curiosity-enhanced memory for incidental faces (Fig. 2). The results indicated a significant interaction between curiosity and the time point of face presentation (*F*_(1,59)_ = 7.86, *P* = 0.007, partial eta squared = 0.118; Fig. 2). Neither the main effect of curiosity (*F*_(1,59)_ = 1.96, *P* = 0.167, partial eta squared = 0.032) nor the main effect of time point of face presentation (*F*_(1,59)_ = 0.46, *P* = 0.498, partial eta squared = 0.008) reached significance. Follow-up one-tailed *t*-tests revealed that, in the early presentation group, recollection estimates for faces were significantly higher for high-curiosity compared with low-curiosity trials (*t*_(29)_ = 2.76, *P* = 0.005, Cohen's *d* = 0.505, mean difference = 3.87, lower = 1.49, upper = ∞). In contrast, for late presentation, we did not find a significant difference in recollection for faces between the high- and low-curiosity condition (*t*_(30)_ = −1.08, *P* = 0.854, Cohen's *d* = −0.193, mean difference = −1.29, lower = −3.33, upper = ∞) (for further analyses, see Supplemental Material, sections 2.1–2.2).

**Figure 2.**
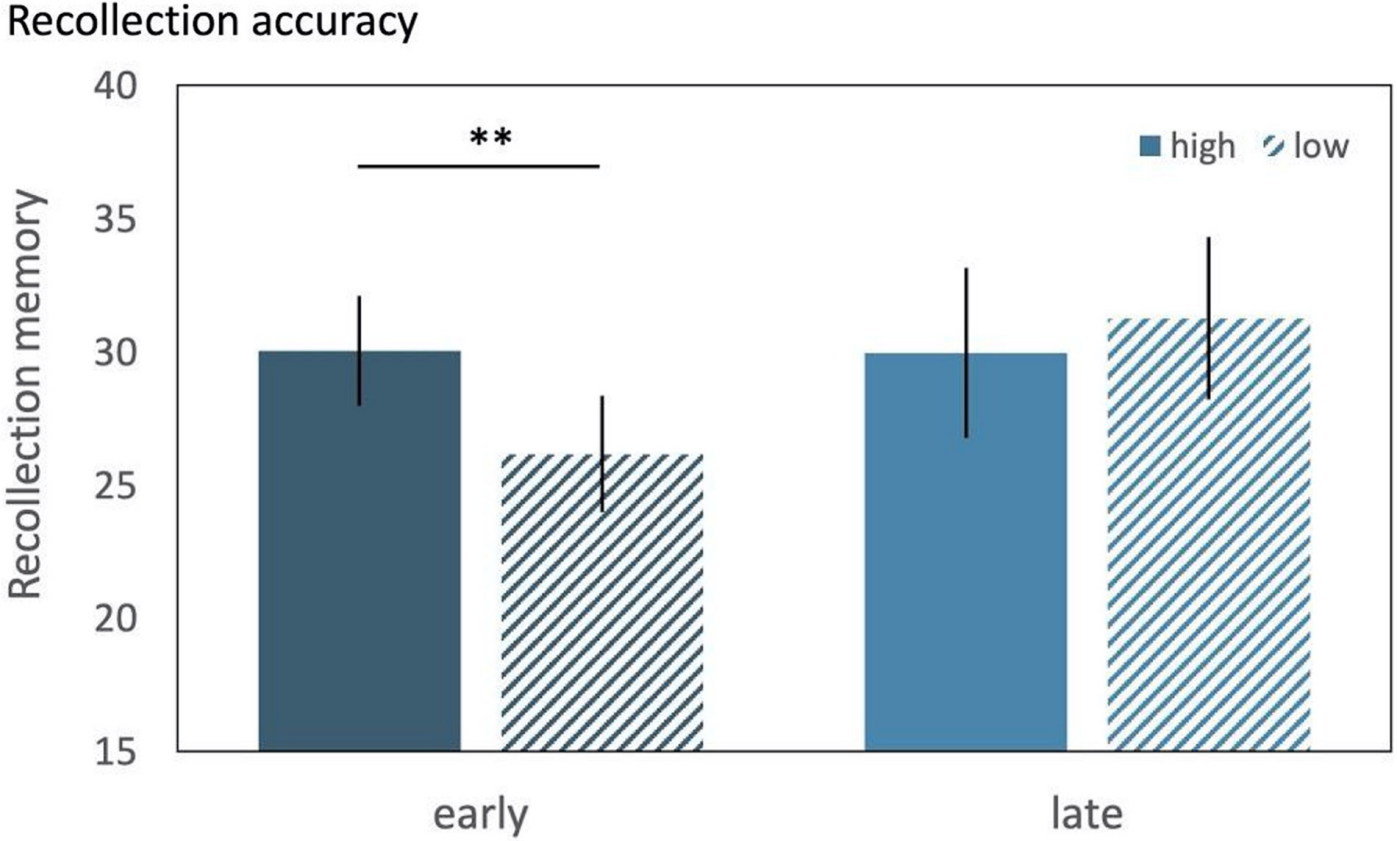
Recollection accuracy for incidental faces for experiment 1. Proportion of correctly remembered faces for the early and late face presentation groups split by high and low curiosity conditions. Results reveal a significant interaction between curiosity state and time point of face presentation on subsequent memory for incidental faces. Participants remembered more faces when in a high-curiosity compared with a low-curiosity state if the face was presented early in the anticipation period. Recollection accuracy for incidental items were as follows: high curiosity early (mean = 30.04%, SD = 11.22), low curiosity early (mean = 26.17%, SD = 12.02), high curiosity late (mean = 29.97%, SD = 17.74), and low curiosity late (mean = 31.26%, SD = 16.95), curiosity-enhanced memory (high—low) early (mean = 3.87%, SD = 7.66) and late (mean = −1.29%, SD = 6.68). Standard error is depicted by a vertical black line. (**) *P* < 0.01. The findings of experiment 1 suggest that high-curiosity compared with low-curiosity states show enhanced memory for incidental information if it is presented in close proximity to the elicitation of curiosity. These effects were specific to recollection, did not generalize across familiarity-based recognition, and could not be explained by performance on the encoding judgments (see Supplemental Material, sections 2.1–2.2).

To investigate whether there was a difference in the way the face stimuli were encoded, we investigated whether RTs of the encoding judgment as a potential index of “alertness” during encoding differed between conditions. Therefore, we ran a two-way mixed effects ANOVA on RTs with curiosity (high vs. low) as within-subjects factor and the time point of face presentation (early vs. late) as between-subjects factor (see Supplemental Table S1 for means (SDs)). Neither the main effects of curiosity (*F*_(1,59)_ = 3.03, *P* = 0.087, partial eta squared = 0.049), time point of face presentation (*F*_(1,59)_ = 1.09, *P* = 0.300, partial eta squared = 0.018) nor their interaction (*F*_(1,59)_ = 3.76, *P* = 0.057, partial eta squared = 0.059) reached significance suggesting that “alertness” potentially did not differ between curiosity and timing of face presentation conditions. However, to further interrogate whether RTs, as a potential index of “alertness,” during encoding had any effects on later memory, we included curiosity-related RT differences during encoding (i.e., high-curiosity RTs—low-curiosity RTs) as a covariate in a two-way mixed effects ANCOVA with curiosity (high vs. low) and time point of face presentation (early vs. late) as factors on recollection memory. Consistent with the ANOVA findings, the interaction between curiosity and time point of face presentation remained when the difference in RTs during high vs. low curiosity encoding was controlled for (*F*_(1,58)_ = 8.23, *P* = 0.006, partial eta squared = 0.124). No other main effects or interactions were significant (curiosity: *F*_(1,58)_ = 2.27, *P* = 0.137, partial eta squared = 0.038; time point of face presentation: *F*_(1,58)_ = 0.22, *P* = 0.644, partial eta squared = 0.004; curiosity-related RT difference: *F*_(1,58)_ = 0.63, *P* = 0.432, partial eta squared = 0.011; curiosity * curiosity-related RT difference: *F*_(1,58)_ = 0.44, *P* = 0.509, partial eta squared = 0.008).

To determine whether participants gave different ratings on the encoding judgment between curiosity conditions and the time point of face presentation, we ran a two-way mixed effects ANOVA on the proportions rated “helpful” (i.e., “yes” responses) with curiosity (high vs. low) and time point of face presentation (early vs. late) as factors. Helpfulness ratings significantly differed with curiosity states (*F*_(1,59)_ = 22.05, *P* < 0.001, partial eta squared = 0.272) but not with time point of face presentation (*F*_(1,59)_ = 0.01, *P* = 0.915, partial eta squared = 0.000). The interaction between curiosity and time point of face presentation was not significant (*F*_(1,59)_ = 0.08, *P* = 0.775, partial eta squared = 0.001). Potentially surprisingly, faces were rated as significantly more helpful during low-curiosity compared with high-curiosity states (see Supplemental Table S2). Due to this significant difference, the curiosity-related difference in helpfulness ratings (i.e., high–low curiosity) was added as a covariate in a two-way mixed effects ANCOVA on recollection accuracy with curiosity (high vs. low) and timing of face presentation (early vs. late) as factors. Importantly, the interaction between curiosity and time point of face presentation remained when the difference in helpfulness ratings during high vs. low curiosity encoding was controlled for (*F*_(1,58)_ = 7.86, *P* = 0.007, partial eta squared = 0.119). No other main or interaction effects were significant (curiosity: *F*_(1,58)_ = 0.84, *P* = 0.364, partial eta squared = 0.014; timing of face presentation: *F*_(1,58)_ = 0.43, *P* = 0.517, partial eta squared = 0.007; curiosity-related helpfulness rating difference: *F*_(1,58)_ = 0.49, *P* = 0.487, partial eta squared = 0.008; curiosity * curiosity-related helpfulness rating difference: *F*_(1,58)_ = 0.27, *P* = 0.604 partial eta squared = 0.005).

In experiment 2 (N = 32) (see Supplemental Material, section 1.1), during the anticipation window an unrelated face replaced the fixation cross after 2, 4, 6, or 8 sec (Fig. 1D) spanning the entire anticipation period. During the presentation of the face, participants had to rate how pleasant they found the face, from 1 (“not at all pleasant”) to 6 (“extremely pleasant”), after which the fixation cross reappeared for the remainder of the anticipation period (either 8, 6, 4, or 2 sec depending on when the face image was presented) (see Fig. 1D). This decision-making judgment was deemed incidental as it was not semantically related to the trivia question. Finally, in experiment 2, we implemented a 1-d delayed surprise memory test, as the experiment served as a pilot experiment for a potential future neuroimaging experiment, and as such, we wished to reduce task demands on individuals on the day of scanning. Participants therefore made a four-point confidence judgment on whether they thought the face was presented during the study phase (see Supplemental Material, section 1.3). The four-point confidence judgment is consistent with previous studies that showed curiosity-enhanced memory for incidental faces in delayed memory tests (Gruber et al. 2014; Stare et al. 2018).

Following up the findings of experiment 1, in experiment 2 we used a linear regression model to determine whether curiosity-enhanced memory of incidental information linearly decreased at larger intervals from the elicitation of curiosity. This model indeed revealed a significant effect of time point on curiosity-enhanced memory (*F*_(1,126)_ = 9.91, *P* = 0.002, *R*^2^ = 0.073; Fig. 3A). Next, to determine at which time point the curiosity-enhanced memory effect was significant, we conducted four follow-up one-tailed t-tests investigating when recognition memory for faces was higher in high-curiosity compared with low-curiosity conditions (i.e., at 2, 4, 6, and 8 sec). This analysis revealed a significant difference at both 2 sec (*t*_(31)_ = 3.06, *P* = 0.002, confidence intervals (5.28 and 26.35), mean difference = 15.8), and 4 sec (*t*_(31)_ = 1.73, *P* = 0.045, confidence intervals (−0.98 and 11.91), mean difference = 5.5), but no significant difference at 6 and 8 sec (*P* = 0.380 and *P* = 0.471, respectively) (Fig. 3B).

**Figure 3.**
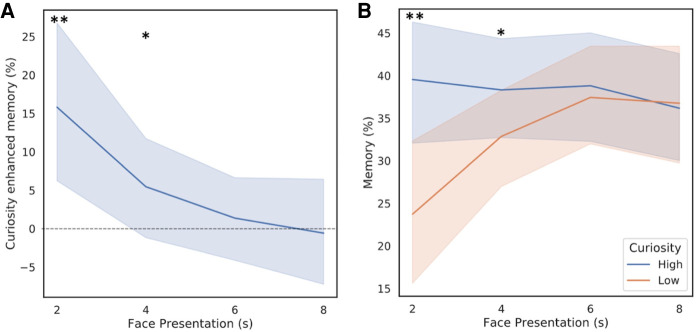
Recognition memory for incidental faces for experiment 2. (*A*) Curiosity-enhanced memory (high–low) at each presentation time point. (*B*) Percentage of correctly recognized faces at each presentation time point, split by high- and low-curiosity conditions. Participants recognized significantly more faces when in a high-curiosity trial if the face was presented early in the anticipation window (at both 2 and 4 sec). (**) *P* < 0.01, (*) *P* < 0.05. Ninty-five percentage confidence intervals are depicted by the shaded area. Recognition memory for incidental items were as follows: curiosity-enhanced memory (high–low) (2 sec: mean = 15.80%, SD = 29.21; 4 sec: mean = 5.54%, SD = 17.89; 6 sec: mean = 1.33%, SD = 16.26; 8 sec: mean = −0.62%, SD = 20.24). For each time point the recognition memory scores were as followed: 2 sec (high curiosity: mean = 39.58%, SD = 21.37; low curiosity: mean = 23.70%, SD = 29.83), 4 sec (high curiosity: mean = 38.81%, SD = 17.38; low curiosity: mean = 32.92%, SD = 18.28), 6 sec (high curiosity: mean = 38.78%, SD = 19.70; low curiosity: mean = 37.53%, SD = 17.93) and 8 sec (high curiosity: mean = 36.18%, SD = 19.66; low curiosity: mean = 36.76%, SD = 22.01). Extrapolating from this data we found that, consistent with experiment 1, incidental curiosity-enhanced memory is largest when presented in close proximity to the elicitation of curiosity (i.e., 2 sec after the presentation of a trivia question), but its magnitude linearly decreased with increasing time interval from the elicitation of curiosity.

To determine whether pleasantness judgment differed across curiosity states or memory performance we ran a 2 (curiosity; high, low) × 2 (memory; hits, misses) repeated measures ANOVA on participants pleasantness ratings. This analysis revealed that pleasantness ratings did not significantly differ across memory (*F*_(1,31)_ = 1.43, *P* = 0.23) or curiosity effects (*F*_(1,31)_ = 1.93, *P* = 0.17). See Supplemental Table S2 for mean pleasantness ratings.

Next, to determine whether this effect was due to participants being more “alert” during high curiosity trials we investigated whether response times (RT) to incidental face stimuli differed across time point, condition and memory performance (see Supplemental Table S1 for mean RTs). A 2 (condition; high curiosity, low curiosity) × 2 (memory; hits, misses) × 4 (time point; 2, 4, 6, and 8 sec) repeated-measures ANOVA was conducted revealing no significant difference in RT score across time point (*F*_(3,48)_ = 1.58, *P* = 0.21), condition (*F*_(1,16)_ = 0.34, *P* = 0.57), memory (*F*_(1,16)_ = 1.07, *P* = 0.32), nor the interactions between these.

Finally, for consistency with our previous analysis and to determine whether our linear regression effect was due to (1) participants being more “alert” during high-curiosity trials (as inferred by RTs) or (2) participants’ degree of pleasantness rating for the faces (as inferred by encoding judgment), we included two additional independent variables to our previous linear regression model. A multiple regression was therefore run to predict curiosity-enhanced memory performance from time point, curiosity-related RT differences (high–low curiosity) and curiosity-related pleasantness rating (high–low curiosity). These variables significantly predicted curiosity-enhanced memory (*F*_(3,126)_ = 4.14, *P* = 0.008, *R*^2^ = 0.069). Importantly, only time point added significantly to the model prediction (*P* = 0.002), whereas curiosity-related RT differences (*P* = 0.280) and curiosity-related differences in pleasantness rating (*P* = 0.209) did not. Collectively, this indicates that only the time point of face presentation significantly predicted curiosity-enhanced memory even when controlling for RT and pleasantness ratings.

Taken together the current work yields several important contributions to our understanding of how curiosity affects memory for incidental information. First, this work provides further evidence that suggests that high-curiosity states relative to low-curiosity states can improve memory of information beyond the target of a person's curiosity. Our results suggest that the constraints of this effect are determined by the temporal proximity of incidental information to the elicitation of curiosity. Second, across both experiments we found early curiosity effects on incidental memory were independent of the nature of the incidental encoding judgment (i.e., how knowledgeable or pleasant participants rated the face). In addition, reaction times for encoding judgments as a potential measure of “alertness” did not predict curiosity-enhanced memory across experiments.

Recent theories on curiosity (e.g., Gruber and Ranganath 2019; Murayama 2019; Sharot and Sunstein 2020) highlight the importance of dopaminergic brain regions in supporting curiosity and curiosity-related memory enhancements. Although we cannot make strong conclusions about whether our findings are a result of increased release of dopamine, there is reason to believe dopamine may play an important role as our results align with predictions from fMRI findings regarding regions innervated by dopamine including the hippocampus, which suggest that the curiosity-related neural activity is somewhat limited in duration, and potentially fitting with a time-course of clearance of curiosity-triggered dopamine release via reuptake across seconds (Düzel et al. 2010; Shohamy and Adcock 2010; Gruber et al. 2014). As fMRI signals in the dopaminergic midbrain have been shown to positively correlate with dopamine release (Knutson and Gibbs 2007; Schott et al. 2008), this pattern of dopaminergic involvement suggests dopamine might be released during the elicitation of curiosity, which benefits hippocampus-dependent memories for incidental information presented in close succession to this release. Speculatively, this provides a plausible neuromodulatory explanation as to why faces presented at larger intervals from the elicitation of curiosity did not benefit from participants heightened state of curiosity. This finding is also consistent with research in the field of reward, that indicates dopaminergic activity scales with high perceived reward, and this predicts incidental memory (Tobler et al. 2005; Murty and Adcock 2014; Stanek et al. 2019).

Perhaps surprisingly, experiment 2 showed that during high-curiosity trials, memory for incidental faces is consistent across all four time points, whereas in low-curiosity trials memory for incidental faces improves at later presentation times (see Fig. 3B; Supplemental Material, section 4.1). Our results are therefore inconsistent with arousal-biased theories (e.g., Mather et al. 2016), which predict that arousal, potentially elicited by high curiosity, would supress attention of irrelevant information (e.g., incidental faces). Instead, our results are consistent with recent literature in the field of reward that showed that early during reward anticipation, memory formation was improved by increased expected reward value (akin to high curiosity) potentially due to a phasic dopamine response, whereas late during reward anticipation, memory formation was enhanced by reward uncertainty reflected by a sustained, ramping of anticipatory dopamine release (Stanek et al. 2019). Notably, the findings by Stanek et al. (2019) might provide a plausible neuromodulatory explanation as to why memory for incidental faces was comparable in high-curiosity trials at all time points as it was supported by potentially both (1) phasic dopamine bursts in the early presentation and (2) sustained anticipatory dopamine ramping in the later presentations. In contrast, low curiosity memory for incidental information would only be facilitated by the later sustained anticipatory dopamine release driven by uncertainty about the correct answer. Our earlier neuroimaging findings showed that individual differences in activation of dopaminergic areas and the hippocampus elicited by curiosity (i.e., during trivia questions) predicted the magnitude of curiosity-enhanced memory for incidental faces (Gruber et al. 2014). Our current findings complement these earlier results suggesting that curiosity-elicited activity in these areas might only enhance memory for incidental faces in close temporal proximity. Despite our findings aligning with previous literature that highlights dopaminergic involvement our interpretations are speculative, therefore the neural investigation of curiosity-triggered modulation of incidental memory require further investigation.

Another important finding was that curiosity-enhanced memory for incidental face information was also robustly seen across two different types of incidental encoding judgments and was not influenced by encoding judgment performance. In experiment 1, participants determined “whether the particular person would be knowledgeable about the trivia topic.” Using the same incidental encoding judgment as in previous trivia studies (Gruber et al. 2014; Galli et al. 2018; Stare et al. 2018; Fandakova and Gruber 2020), we replicated and extended the previous findings in showing that incidental curiosity-enhanced memory is specific to the temporal proximity to curiosity elicitation. Although the faces are incidental in the sense that they did not provide any meaningful information relating to the trivia, one could argue that the faces are task-relevant due to the nature of the decision-making judgment. Importantly, curiosity-enhanced memory for incidental faces was still evident with an encoding judgment that is not semantically associated with the trivia stimuli (i.e., rating the pleasantness of the incidental face image in experiment 2). Taken together, these findings suggest high-curiosity states can improve memory of information beyond the target of a person's curiosity, even when that information is completely incidental to the topics that piqued an individual's curiosity.

In conclusion, our findings provide a better understanding into how curiosity enhances memory for incidental information, suggesting that the elicitation of curiosity is key. However, future studies should examine the role of curiosity on incidental memory for a broader range of stimuli and with different approaches to elicit curiosity. The impact of such work could be applicable to a wide range of areas and would need to be tested in the real world (for example, in news or education). This may be particularly pertinent given the current pandemic in which the need to disseminate rapidly updating policies and educate the public on disease and health, has never been more apparent. As such, understanding when to present critical information that should be remembered is pivotal. Our results indicate it is important that the to-be-enhanced incidental information is presented as early as possible when curiosity is sparked.

## Supplementary Material

Supplemental Material
