# Supplementary material for: Temporal proximity to the elicitation of curiosity is key for enhancing memory for incidental information
Source: Learn Mem. 2021 Feb;28(2):34–9. doi: 10.1101/lm.052241.120 (PMC7812865; doi:10.1101/lm.052241.120)
Supplement: Supplemental Material [file supp_28_2_34__index.html]

Supplemental Material 

# Temporal proximity to the elicitation of curiosity is key for enhancing memory for incidental information

## Supplemental Material

- Supplementary\_Materials.docx
