# Supplementary material for: Temporal proximity to the elicitation of curiosity is key for enhancing memory for incidental information
Source: Learn Mem. 2021 Feb;28(2):34–9. doi: 10.1101/lm.052241.120 (PMC7812865; doi:10.1101/lm.052241.120)
Supplement: Supplemental Material [file supp_28.2.34_Supplementary_Materials.docx]

**Supplementary Materials**

**1. Experiment 1 - supplementary materials and methods**

**1.1 Participants**

A total of 77 healthy undergraduate students from the UC Davis community were recruited. Participants were native English speakers and had normal or corrected to normal vision. The participants received two hours of course credit and were tested in groups of two to seven participants. Participants were randomly assigned to either the ‘Early’ or ‘Late’ presentation condition. In total, data of 16 participants were excluded from the analysis due to the following reasons: 6 participants because they showed poor memory performance for the face images (i.e., < 10 % recollection accuracy), 4 participants due to technical problems, 3 participants because they did not get enough trials in either the high- or low-curiosity condition during the screening phase, and 3 participants because they expected a memory test or took part in a previous experiment involving a trivia paradigm. Consequently, the final ‘Early’ and ‘Late’ presentation group included 30 and 31 participants, respectively. The average age across these remaining 61 participants was 19.5 years (range = 18 – 24) with 45 females. Ethical approval was obtained from the UC Davis Institutional Review Board and informed consent was obtained from each participant prior to taking part in the experiment.

**1.2 Materials**

The materials were identical with our earlier work on curiosity (Gruber et al. 2014; Stare et al. 2018).

Trivia questions and answers. Stimuli were 375 trivia questions with corresponding answers used in previous publications to elicit different levels of curiosity (Gruber et al. 2014). The trivia stimuli are available online at OSF (<https://osf.io/he6t9/>). These trivia questions corresponded to a variety of categories: history/geography, movies/TV, music, nature, science, space, sports, food and other miscellaneous facts. Because we wanted participants to learn the answers during the study phase, the pool only included trivia questions for which the answers were likely to be unknown to the majority of participants. During the screening phase participants selected 112 trivia items (56 high curiosity, 56 low curiosity) from the 375 questions. The allocation of trivia questions to high- or low-curiosity conditions was therefore dependent on participants’ ratings during the screening phase.

Faces. As in our earlier work (Gruber et al. 2014; Stare et al. 2018), a pool of photographs of emotionally neutral faces with naturalistic backgrounds was used in the experiment (stimuli were a subset of face stimuli originally used in: Bialleck et al. 2011). In Experiment 1, 168 face stimuli were divided into three sets (56 stimuli each) and these sets were counterbalanced across participants for the following three trial types: faces presented in high- and low-curiosity conditions during the study phase and faces that served as new stimuli during the recognition test phase.

Screening phase: Because the level of curiosity elicited by different trivia questions has been shown to considerably vary between participants, we used participants’ ratings to sort trivia questions into participant-specific high- and low-curiosity categories (56 questions each). Trivia questions were randomly selected from a pool of 375 trivia questions and were consecutively presented. Each trivia question was presented for 4 s. After the presentation of a trivia question, participants had to give two self-paced ratings on six-point scales (Fig 1A). First, they had to rate how confident they were that they knew the answer to a trivia question (extreme points: 1 = “I am confident that I do not know the answer” and 6 = “I am confident that I know the answer”). Second, participants rated their level of curiosity about the answer to a trivia question (extreme points: 1 = “not curious at all” and 6 = “very curious”). If participants did not indicate that they knew the answer to a trivia question (i.e., they did not give a 6 response on the answer confidence rating), trivia questions with responses 1-3 of the curiosity rating were allocated to the low-curiosity condition and responses 4-6 to the high-curiosity condition. Responses were made on a computer keyboard and responses 1-3 were made with participants’ left hand and 4-6 with participants’ right hand. The screening phase lasted until 56 trivia questions were allocated for each curiosity condition. After a response was given for the second rating, an inter-trial cross hair was presented with a duration of 1 s.

**1.3 Recognition memory test for incidental items**

For each face, participants were instructed to press one of six buttons on a computer keyboard: R, 5, 4, 3, 2, and 1 (representing the following responses: R = “confidently remembered with specific details”, 5 = “confidently familiar without any details”, 4 = “unconfident familiar”, 3 = “guessing”, 2 = “unconfident new”, 1 = “confident new”). Participants were encouraged to try to give a response as accurately and quickly as possible.

Recollection of incidental faces was computed by subtracting false alarms (i.e., participants incorrectly ‘*remembered’* an item as old (pressed R) when it was ‘new’) from hits (i.e., participants correctly pressed R indicating they *remembered* seeing the item). To account for response non-independence, familiarity was computed by conditionalizing on the opportunity to make a ‘familiar’ response (i.e., collapsed across 5 = ‘confident familiar’ and 4 = ‘unconfident familiar’ responses) when a ‘remember’ response was not made (see Libby et al. 2013). That is:

Familiarity _old items_ = Familiarity (‘5’ & ‘4’ response)_old items_  ∕ (1 − Remember (‘R’ response) _old items_)

Familiarity _new items_ = Familiarity (‘5’ and ‘4’ response)_new items_ ∕ (1 − Remember (‘R’ response) _new_)

Overall familiarity, correcting for false alarms is then estimated as:

Familiarity = Familiarity _old items_ − Familiarity _new items_

The findings did not differ if familiarity estimates were only restricted to ‘5’ (‘confident familiar’) responses instead of collapsing across confidence (‘i.e., 5’ and ‘4’ responses). Results for recollection and familiarity will be analysed separately.

**2. Experiment 1 - supplementary results**

**2.1 Results for familiarity-based and high-confidence recognition**

To investigate the specificity of the significant interaction for recollection memory and curiosity (reported in the main text), we further interrogated familiarity-based and high-confidence recognition with two separate two-way mixed measures ANOVAs with curiosity (high vs. low) as within-subjects factor and timing of face presentation (early vs. late) as between-subjects factor. The ANOVA on familiarity estimates (see supplementary materials and methods) showed no significant main effects of curiosity (F(1,59) = 0.05, p = .820, partial eta squared = .001) and timing of face presentation (F(1,59) = 0.07, p = .795, partial eta squared = .001). The interaction between curiosity and timing of face presentation was also not significant (F(1,59) = 0.51, p = .478, partial eta squared = .009).

In addition, the ANOVA on high-confidence recognition accuracy (i.e., Hits ( ‘Remember’ & ‘5’ [Confident familiar] collapsed) – False Alarms (‘Remember’ & ‘5’ [Confident familiar] collapsed) also showed no significant main effects of curiosity (F(1,59) = 1.55, p = .218, partial eta squared = .026), timing of face presentation (F(1,59) = 0.03, p = .866, partial eta squared = .001), and no significant interaction (F(1,59) = 1.41, p = .241, partial eta squared = .023).

**2.2 Follow-up t-tests for recollection of incidental faces within high- and low-curiosity conditions separately**

To follow up the two-way mixed effects ANOVA reported in the main text, two independent-samples t-tests were carried out to investigate the effect of presentation time (early vs. late) on incidental face memory within high- and low-curiosity trials separately. We found that early vs. late timing of face presentation did not differ within the high- or low-curiosity conditions (high curiosity: t(59) = 0.18, p = .99, Cohen’s d = 0.005, mean difference = 0.07, lower = -7.56, upper = 7.7; low curiosity: (t(59) = -1.35, p = .18, Cohen’s d = -0.35, mean difference = -5.09, lower = -12.64, upper = 2.46).

**3. Experiment 2 - supplementary materials and methods**

**3.1 Participants**

Ethical approval was obtained from the School of Psychology ethics committee at Cardiff University. A total of 39 healthy undergraduate students with normal to corrected vision were recruited for this study. Informed consent was sought prior to taking part in the behavioural experiment. The participants received a monetary reward of £18 or 3h of course credit. In total, 7 participants were excluded from the analysis due to the following reasons; 2 due to technical problems, 3 participants because they did not get enough trials in either the high or low condition during the screening phase, and 2 due to failure to comply with the instructions. No participants were excluded due to poor memory performance. Consequently, the final group included 32 participants. The average age for this group was 20.6 years (range = 18 – 31) with 24 females.

**3.2 Materials**

Trivia questions and answers. Stimuli were 294 trivia questions with corresponding answers pooled from a larger subset (N = 375) of trivia questions used in Experiment 1. The 294 questions were selected if they were considered appropriate for a UK-based population. The trivia stimuli are available online at OSF (https://osf.io/he6t9/). That is, questions specifically targeted towards non-British populations were excluded from the final list. During the screening phase participants selected 128 trivia items (64 high curiosity, 64 low curiosity) from the 294 questions. The allocation of trivia questions to high- or low-curiosity conditions was therefore dependent on participants’ ratings during the screening phase.

Faces. As in Experiment 1 a pool of photographs of emotionally neutral faces was used in the experiment (stimuli were a subset of face stimuli originally used in Bialleck et al. 2011). In this experiment, a pool of 196 face stimuli were pseudo-randomly divided into the following three sets: faces presented in high (N=64) and low (N=64) curiosity conditions during the study phase and faces that served as new (N=64) stimuli during the test phase. The pseudo-random parcellation was to ensure an even number of males and females; 32 males and 32 females were present in each set.

We also presented a naturalistic background scene throughout the entire trial window. As this experiment was a pilot for a neuroimaging design, the purpose of the background images were to help train pattern classifiers to distinguish between our experimental conditions (consistent with our previous work, e.g., Gruber et al. 2016). Note, these background scenes are not relevant to the behavioural analysis in the current study.

Screening Phase

Screening phase was identical to Experiment 1, however 64 high-curiosity and 64 low-curiosity trials were selected (N = 128 in total) (Fig 1A). Therefore, the screening phase lasted until 64 trivia questions were allocated for each curiosity condition (on average 45 minutes).

**3.3 Recognition memory for incidental faces**

The 4-point confidence judgement included 1 = “confident old”, 2 = “unconfident old”, 3 = “unconfident new” and 4 = “confident new” responses. Participants were encouraged to give a response as quickly and accurately as possible. Recognition memory accuracy for incidental faces was computed by subtracting false alarms (i.e., when participants incorrectly recognized an item as ‘old’ (pressed 1 = ‘confident old’ or 2 = ‘unconfident old’) when it was ‘new’) from hits (i.e., when participants correctly pressed 1 (‘confident old’) or 2 (‘unconfident old’) indicating they had seen the item before.

**4. Experiment 2 - supplementary results**

**4.1 Additional linear regression for high- and low-curiosity memory separately**

To follow up the linear regression reported in the main text that showed that curiosity-related memory enhancements for faces showed a linear relationship with the timepoint of face presentation, two additional regressions were run to interrogate the effect of timepoint within high and low curiosity separately. These revealed no significant difference of timepoint on high-curiosity memory performance (F(3,93) = 0.66, p = .42), but a significant difference for low-curiosity memory performance (F(3,93) = 6.55, p < .001). Visual inspection indicates that this timepoint difference in the low-curiosity condition is capturing a linear trend (Fig 3B), showing lower memory performance in early time points (2 and 4 s) and increased memory performance for later time points (6 and 8 s). This suggests that curiosity-enhanced memory (i.e., high – low curiosity) was driven by lower memory performance in ‘low-curiosity’ conditions during earlier presentation of incidental information, and comparable memory performance for high- and low-curiosity conditions in later presentation timepoints.

Table S1. Reaction times (RTs) on incidental faces split by condition for both experiments.

| **Experiment 1** | | | | **Experiment 2** | | | |
| --- | --- | --- | --- | --- | --- | --- | --- |
| Curiosity | Group | Mean (ms) | SD | Curiosity | Timepoint | Mean (ms) | SD |
| High | Early | 1461 | 431 | High | 2 | 1375 | 539 |
| Low |  | 1413 | 443 | Low |  | 1291 | 705 |
| High | Late | 1337 | 300 | High | 4 | 1350 | 551 |
| Low |  | 1339 | 295 | Low |  | 1365 | 499 |
|  |  |  |  | High | 6 | 1462 | 435 |
|  |  |  |  | Low |  | 1417 | 496 |
|  |  |  |  | High | 8 | 1235 | 608 |
|  |  |  |  | Low |  | 1250 | 518 |

*Footnote: SD = standard deviation.*

Table S2. Encoding judgements on incidental faces split by condition for both experiments

| **Experiment 1**  **(knowledgeable?)** | | | | **Experiment 2**  **(pleasant?)** | | | |
| --- | --- | --- | --- | --- | --- | --- | --- |
| Curiosity | Group | Mean | SD | Curiosity | Timepoint | Mean | SD |
| High | Early | 45.417 | 13.418 | High | 2 | 2.83 | 1.11 |
| Low |  | 52.143 | 15.031 | Low |  | 2.57 | 0.80 |
| High | Late | 44.585 | 16.956 | High | 4 | 3.06 | 0.88 |
| Low |  | 52.189 | 16.267 | Low |  | 2.52 | 0.76 |
|  |  |  |  | High | 6 | 3.09 | 0.98 |
|  |  |  |  | Low |  | 2.63 | 0.96 |
|  |  |  |  | High | 8 | 2.78 | 1.09 |
|  |  |  |  | Low |  | 2.51 | 0.92 |

Footnote: Mean response in Experiment 1 refers to the average proportion that a participant responded ’yes’ a face/person depicted would be knowledgeable about the question. Mean response in Experiment 2 refers to the average response (1-6 pleasantness rating) that a participant responded to how pleasant they found the face/person.
